# Biomechanical Analysis of the Use of Stems in Revision Total Knee Arthroplasty

**DOI:** 10.3390/bioengineering9060259

**Published:** 2022-06-19

**Authors:** Bernardo Innocenti, Edoardo Bori, Silvia Pianigiani

**Affiliations:** BEAMS Department, Université Libre de Bruxelles, Av. F. Roosevelt, 50 CP165/56, 1050 Bruxelles, Belgium; edoardo.bori@gmail.com (E.B.); silvia.pianigiani84@gmail.com (S.P.)

**Keywords:** revision TKA, stem, biomechanics, micromotions, bone stress, risk of fracture

## Abstract

Adequate fixation is fundamental in revision total knee arthroplasty; consequently, surgeons must determine the correct set-up for each patient, choosing from numerous stem solutions. Several designs are currently available on the market, but there are no evidence-based quantitative biomechanical guideline yet. Therefore, several stems were designed and analyzed using a previously-validated finite-element model. The following parameters were studied: stem design characteristics (length and shape), added features (straight/bowed stem), fixation technique, and effect of slots/flutes. Bone stress and Risk of Fracture (RF) were analyzed in different regions of interest during a squat (up to 120°). For the femoral stem, the results indicated that all parameters influenced the bone stress distribution. The maximum von Mises stress and RF were always located near the tip of the stem. The long stems generated stress-shielding in the distal bone. Regarding the tibial stem, cemented stems showed lower micromotions at the bone-tibial tray interface and at the stem tip compared to press-fit stems, reducing the risk of implant loosening. The results demonstrated that anatomical shapes and slots reduce bone stress and risk of fracture, whereas flutes have the opposite effect; no relevant differences were found in this regard when alternating cemented and press-fit stem configurations. Cemented tibial stems reduce antero-posterior micromotions, preventing implant loosening.

## 1. Introduction

The increasing number of patients undergoing primary Total Knee Arthroplasty (TKA) (720,000 in the USA during 2010) has been accompanied by a similar increase in the number of revision total knee arthroplasties (70,000 in the USA during 2011) [1,2,3]; furthermore, one study estimated that the overall incidence of primary and revision TKA will grow by 174% and 600%, respectively, between 2005 and 2030 [4].

Although it is widely recognized that the long-term results of primary TKA have been excellent (with reported survival rates of 90–95% at 10 to 15-year follow-up [5,6,7]), revision TKA-related studies evaluated survival rates from 71% to 98% (with varying definitions of failure [6,8,9,10,11,12]).

The main reason for such reduced performance is related to the fact that revision total knee arthroplasty is a complex operation that requires the surgeon to overcome a different set of reconstructive issues, including metaphyseal bone loss, proper fixation of the implant, and ligamentous instability [5,6,7]. In order to succeed, therefore, revision knee arthroplasty requires careful preoperative assessment, accurate planning, and excellent surgical technique. In addition to the difficulty of revision TKA surgical operation itself, determining the most suitable implant type for each patient among a wide array of different products [7,13] is another complex but fundamental step. Among the different options, many treatment strategies and design features could be applied in terms of intramedullary stems, and several solutions are currently available on the market; most of the major TKA manufacturers provides modular revision knee systems and a wide range of stems, with variable lengths, designed to engage in the metaphysis or in the diaphysis and thus enhance fixation in revision situations [14,15]. Intramedullary jig systems are also usually provided to help make accurate revision bone cuts.

Even if it is well known that different stem shapes, lengths, and sizes imply different bone stresses and thus eventual changes in implant stability (which could worsen long-term implant performance [16,17,18,19,20,21,22]), no evidence-based biomechanical guideline is currently available to rely on when choosing stems for a specific clinical situation.

Nowadays, the use of numerical simulations in orthopedic research allows for analyzing different clinical configurations and performing experimental tests in a less invasive and expensive way [23,24]. Among the different approaches, finite element analysis (FEA) is a valid technique to estimate and compare stresses in the bone, providing a valid tool for the interpretation of its interaction with the implant [23,24].

The aim of this study was to analyze, using a validated finite element model, the changes in femoral and tibial bone stress induced by the insertion of different stem designs; a range of features such as length, shape, slot, and presence/absence of cement were thus analyzed in correlation with bone stress, risk of fracture, and implant fixation.

For all the analyzed configurations, the outputs were evaluated in different regions of interest and compared with a “control model” in which no stem was adopted.

## 2. Materials and Methods

All the steps used for the development of the model, from geometry definition, material models and properties, loads, boundary conditions, and mesh, were performed in strict agreement with the previously validate finite element model [22,25,26].

In detail, the model includes the following features:

### 2.1. Geometry

The virtual 3D geometries of the tibia and of the femur were obtained starting from computed tomography (CT) images of the 3D models of the standardized right side femur and tibia [27]; such models are widely used for biomechanical experiments and computer simulations [28,29,30,31]. These geometries include cortical bone, cancellous bone, and intramedullary canal.

In terms of soft tissues, the lateral collateral ligament (LCL) and both the anterior and posterior medial collateral ligaments (aMCL and pMCL) were incorporated into the model. The origin and the insertion points of each collateral ligament were determined according to the definition reported in the literature and used for the development of other similar models [26,32,33,34].

A GEMINI^®^ SL^®^ Fixed Bearing PS (Waldemar LINK GmbH & Co. KG, Hamburg, Germany) was adopted and implanted in the femoral and tibial bone model, following the surgical protocol provided by the manufacturer. According to the dimensions of the bones, a right-side medium size was chosen for both the femoral and tibial components. A polyethylene thickness of 8 mm was considered adequate for the insert used in this study.

Being the study focused on revision TKA, the control configuration was defined as the one with primary TKA without stem (instead of considering the native joint) as this configuration usually precedes the revision implant; the prosthesis was then positioned in a neutrally aligned configuration, inserting the femoral and tibial component orthogonally to the respective mechanical axis [35,36].

### 2.2. Analyzed Femoral Stem Configurations

**Figure 1:**
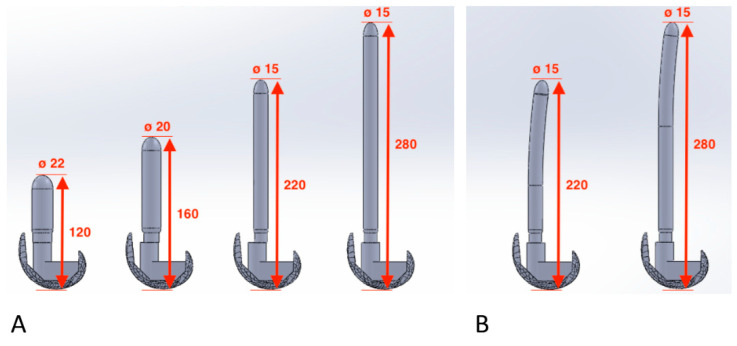
Design of the stems analyzed in the present study with relative length and chosen diameter: (**A**) Straight stems; (**B**) Bowed stems. All the measurements are expressed in mm.

**Figure 2:**
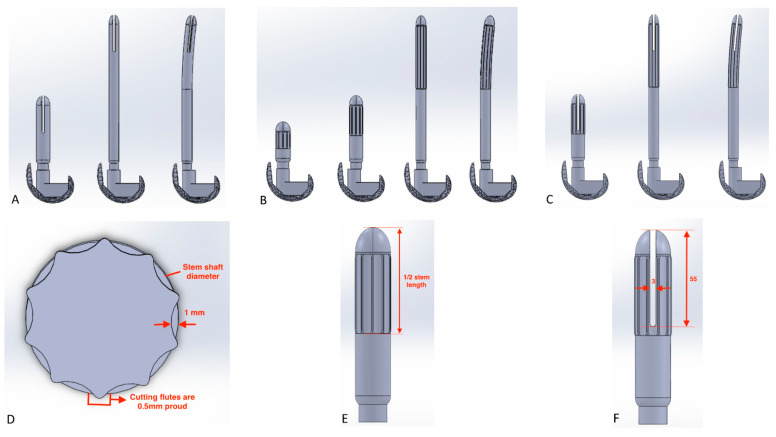
Design of the different stems analyzed in the present study with different features: (**A**) Slotted stems—from the left to the right, 160 mm stem, 280 mm straight, and bowed stems; (**B**) Fluted stems—from the left to the right, 160 mm stem, 280 mm straight, and bowed stem; (**C**) Fluted and slotted stems—from the left to the right, 160, 280 mm straight, and 280 mm bowed stems; (**D**) Design of the fluted stem, transversal cut; (**E**) Lateral view of the fluted stem; (**F**) Lateral view of the slotted stem. All the measurements are expressed in mm.

After an inquiry on currently available products, different designs for femoral stems were developed and analyzed (Figure 1 and Figure 2).

Four parameters were studied:

- Length (L)–Diameter (ϕ) [mm] (Figure 1):

Following clinical practice (and similarly to the study carried out by Soenen et al. [30]), for each length, a specific diameter was chosen to fit in the femoral bone in the best way (resulting in an inverse proportionality ratio between stem diameters and lengths), ensuring stability yet avoiding deep penetration in the cortical bone; the cross-section of the bone indeed is not constant along its length (as femoral bone shaft narrows in the diaphysis) so an overpenetration of this tissue could lead to fractures. Four lengths (and respective diameters) were investigated in this study: 120 mm (with a diameter of 22 mm), 160 mm (diameter 20 mm), 220 mm (diameter 15 mm), and 280 mm (diameter 15 mm).

- Shape:

Two shapes were investigated, namely straight and bowed (anatomical); the bowed configurations were defined only for the length of 220 and 280 mm (Figure 1);

- Cross-section:

For lengths of 120, 160, and 280 mm, stems with a fluted cross-section were also studied (Figure 2): the stem shaft diameter was set equal to the reamer and it decreased by 1 mm in the center of the flutes (Figure 2), which were in turn 0.5 mm proud from the diameter. The fluted cross-sections were not applied to the whole length of the stem, but only to half of it (Figure 2);

- Stem-end:

For long stems (160, 280 mm), the presence of a slot at the tip of the stem was also investigated: the slot was designed to be 3 mm thick and 55 mm long (as reported in Figure 2).

The different parameters were studied separately in order to highlight the effect of each feature on the femoral bone mechanics.

### 2.3. Analyzed Tibial Stem Configurations

**Figure 3:**
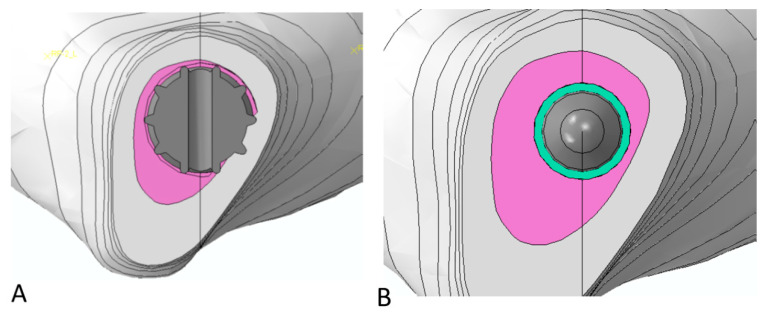
Interaction bone/stem: transversal section view of the area nearby the stem tip: (**A**) Press-fit stem engagement into the cortex; (**B**) cemented tibial stem; a cement layer of 1 mm (in light blue) was considered around the stem.

As was done for the femoral stem, a review of available products was conducted and two lengths of tibial stems were finally designed and analyzed: a short and a long one, respectively, with a length of 90 mm and 170 mm. For every configuration, stems were implanted in the tibia both with a press-fit and a cemented fixation approach; a 1 mm thick cement mantle was implemented in the latter arrangement, whereas for the press-fit stem, a cortex engagement of three teeth was considered acceptable to define the stem positioning and diameter (Figure 3) [30].

### 2.4. Material Models and Properties

In order to model the materials for all the components considered, linear elasticity was applied in agreement with the previous literature studies [26,28,30,35,37,38,39]; as this study does not focus on the analysis of the bone-implant interface from a microscopical point of view, no porous model was considered for the bone material.

Cortical bone was thus considered transversely isotropic with the following materials properties: E1 = E2 = 11.5 GPa, E3 = 17.0 GPa, ν12 = 0.51, and ν23 = ν31 = 0.31 [26,30,40,41] (the third axis was set parallel to the anatomical axis of the femur and of the tibia).

Cancellous bone was then considered linear isotropic with the following material properties: E = 2.13 GPa, ν = 0.3 [37,39,42].

In agreement with previously validated numerical models, the medial and collateral ligament were modeled as beams with specific mechanical properties (LCL: E = 0.111 GPa, ν = 0.45; aMCL and pMCL: E = 0.196 GPa, ν = 0.45 [26,28,32,33,40,42]), cross-sectional area (LCL = 18 mm^2^; aMCL and pMCL = 14 mm^2^ [32,33,43]), and an initial preload (LCL = 0.05; aMCL = 0.04; pMCL = 0.03 [26,28,35,40,42]).

A cobalt-chromium alloy (CoCr), a titanium alloy (Ti-6Al-4V), and an ultra-high-molecular-weight-polyethylene (UHMWPE) were respectively used for the femoral component, tibial tray, and tibial insert; as with the previous study, homogeneous isotropic models were selected for these materials [16,17,43] and their properties were the following: for CoCr: E = 220 GPa, ν = 0.3 [25,32]; for titanium: E = 117 GPa, ν = 0.3 [30,35]; and for UHMWPE: E = 0.685 GPa, ν = 0.4 [28,30,40,42].

A cement layer with a constant in-bone penetration of 3 mm (based on a test of different cementing techniques [44,45,46]) was applied at the interface between the cut surfaces of the femur and the femoral component and between the tibial bone cut surfaces and the tibial component; the material adopted for the cement was set as homogeneous and isotropic with E = 2.62 GPa and ν = 0.3 [26,40,42].

A coefficient of friction of 0.04 was applied for the interaction between the femoral component and the tibial insert, whereas 0.6 was considered for the interaction between the bone and the implant [26,35,39]. A fixed constraint, enabling no relative motion, was then imposed between the stem and the implant.

### 2.5. Load and Boundary Conditions

Loads were obtained from a previously published validated model and were aimed at reproducing compression and shear forces applied on the knee joint [26,28,32,35,47]; a squat activity of up to 120° of flexion was then simulated and the maximum forces found were noted and then applied to each configuration. In detail, a compressive force of 1096 N was applied on the lateral plateau of the insert while a compressive force of 1057 N was applied on the medial plateau. Additionally, a postero-anterior force of 157 N was applied on the posterior surface of the cam to simulate the post-cam engagement.

In the models with femoral stems, the femoral head was fixed via constraint, and the forces were applied on the distal femur; for the models with tibial stems, the distal part of the tibia was considered fixed and the forces were applied on the proximal extremity.

### 2.6. Finite Element Analysis

Quadratic tetrahedral elements with element sizes between 1.5–4 mm were used for the mesh of all the components of the models. To reduce the discretization error, a convergence test was performed to check the selected element size mesh quality for every region of the model. Abaqus/Explicit version 6.13-1 (Dassault Systèmes, Vélizy-Villacoublay, France) was used to perform all the finite element simulations.

For each model and configuration, the average von Mises stress was computed on different Regions of Interest (ROI) in the femoral and tibial bone (see Section 3). In detail, 15 ROIs with a length of 20 mm were defined along the anatomical axis of the femur, starting from a plane placed 200 mm proximally from the distal plane. For the tibia, 22 ROIs were defined starting from the bone cut with a thickness of 10 mm. The outputs were then used to compare the different models among each other and with the control one. Similar to the previous study [30], for the analysis of the femoral stems, the risk of fracture (RF) was also calculated as the ratio between the maximum principal strain in the femur shaft (either compressive or tensile) and the corresponding ultimate strain; for the analysis of the tibial stem, implant micromotions were also investigated.

## 3. Results

### 3.1. Analysis of Femoral Stem

**Figure 4:**
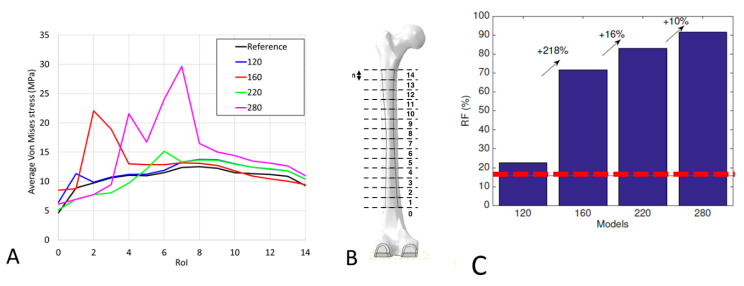
Average von Mises stress for the reference model and for different lengths of straight stem configurations: (**A**) Behavior of the stress in the regions of interest considered for the different configurations; (**B**) Regions of interest analyzed; (**C**) Risk of fracture calculated for each configuration. The red dotted line in the figure reports the risk of fracture for the reference model. The arrows represent the increment in Risk of Fracture compared to the previous configurations.

For all regions of interest analyzed, the average von Mises stress and the risk of fracture for the control model and four different stem lengths (120, 160, 220, and 280 mm, all of which straight model with no additional features) are shown in Figure 4. It is then evident that the insertion of a stem always induces an increase of the bone stress, hence the risk of fracture as well.

**Figure 5:**
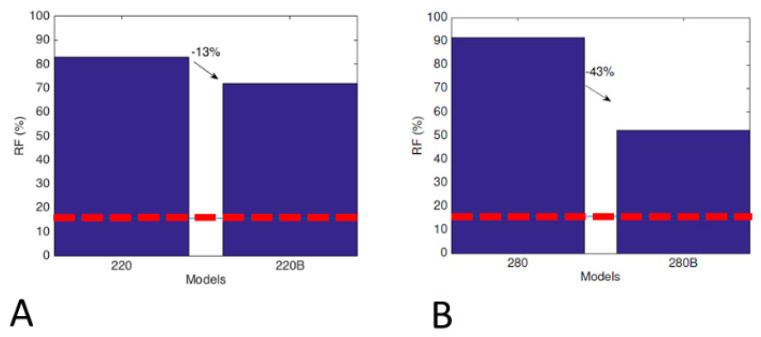

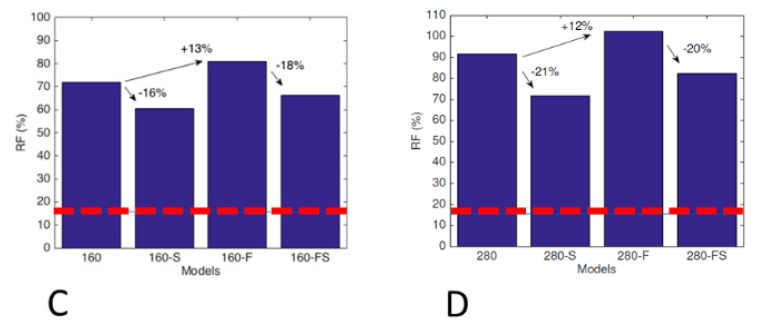
Risk of fracture for several configurations analyzed in the case of femoral stem: (**A**) 220 mm straight (220) and bowed (220B); (**B**) 280 mm straight (280) and bowed (220B); (**C**) stem of 160 mm straight (160), with slot (160−S), fluted (160−F), and fluted with slot (160−FS); (**D**) stem of 280 mm straight (280), with slot (280−S), fluted (280−F), and fluted with slot (280−FS).

Figure 5 reports the RF for several configurations analyzed: in detail, Figure 5A is related to 220 mm straight and bowed stems, Figure 5B relates to the 280 mm straight and bowed ones and, finally, Figure 5C and Figure 5D concern 160 mm and 280 mm straight stems, respectively, with slot, fluted, and fluted with slot.

In all cases involving a bowed stem, the peak of stress value is lower and, consequently, the risk of fracture is lower as well; such reduction of stress and RF is even greater for longer stems, and the presence of a slot always reduces these values even more. Contrariwise, a fluted stem induces an increase in the peak of bone stress and of the relative risk of fracture.

### 3.2. Analysis of Tibial Stem

**Figure 6:**
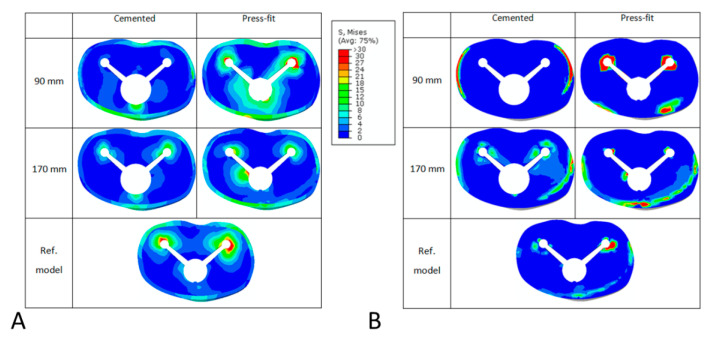
Graphical overview of the von Mises stress (**A**) and of the contact pressure (**B**) at the tibial-bone interface for cemented and press-fit configurations, for stems of 90 mm or 170 mm in length, compared with the reference model in which no stem is present.

To qualitatively highlight the effect of the different parameters investigated, Figure 6 reports a graphical overview of the von Mises stress (Figure 6A) and of the contact pressure (Figure 6B) at the tibial-bone interface for cemented and press-fit configurations, for short and long stems compared to the reference model. The results highlight that the stress distribution in cemented configurations has lower values than the one in press-fit and control models, and that the contact pressure found is also lower; meanwhile, the use of a press-fit stem appears to induce an increment in bone stress and contact pressure at the bone implant interface (especially in case of short stem).

**Figure 7:**
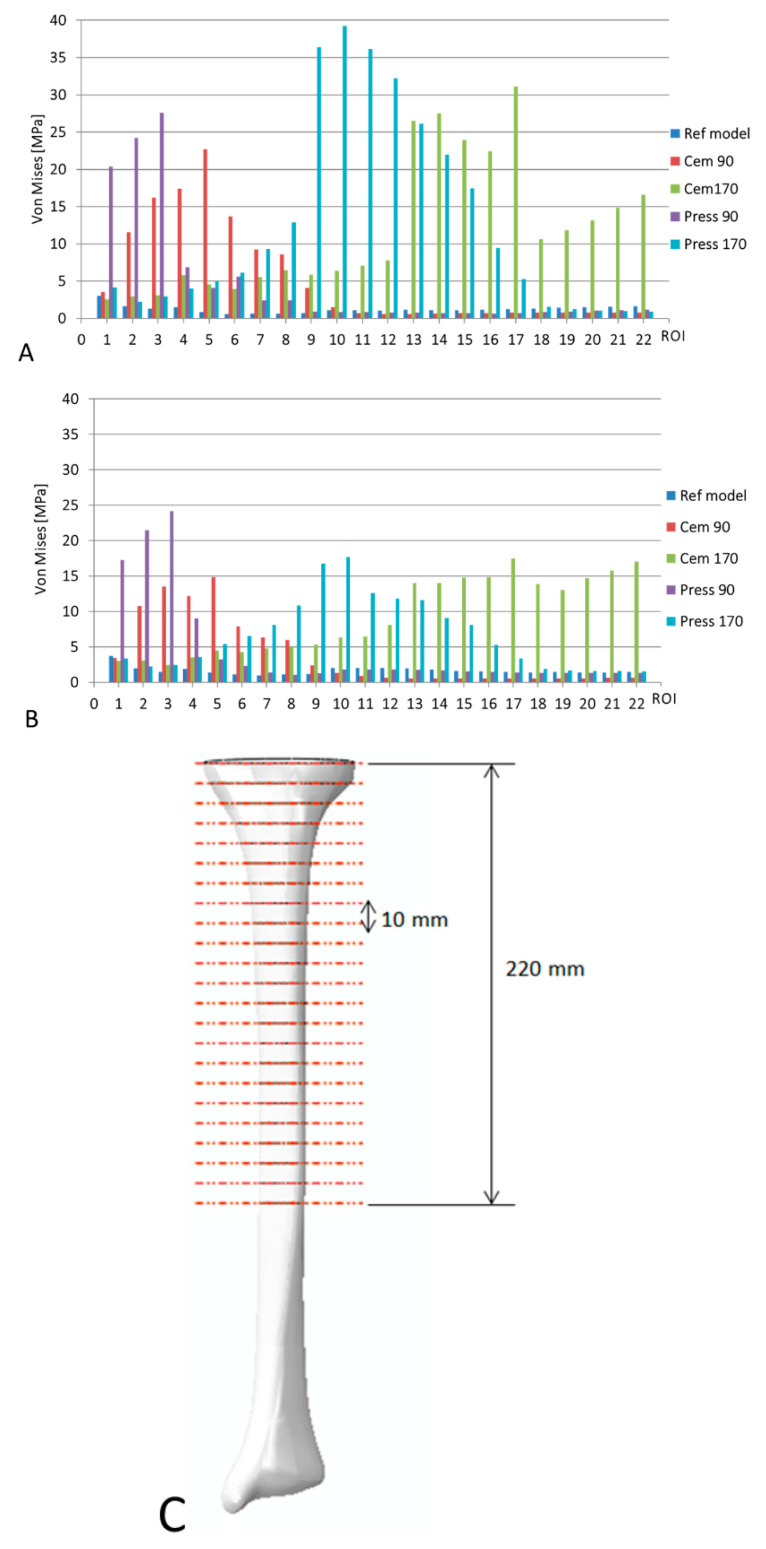
Average von Mises stress estimated in the medial (**A**) and lateral (**B**) side for the different regions of interest (**C**) in the case of the tibial stem. The results of the reference model are also reported in the figure.

Quantitatively, Figure 7 reports the average von Mises stress in the different regions of interests (also illustrated in the figure) for the medial and lateral side. Generally, the stress is higher on the medial side, coherent with the load conditions applied. In agreement with the previous results, the press-fit configurations show higher values than the cemented configurations and short stems induce lower stresses compared to the longer stem for both cemented and uncemented models.

**Figure 8:**
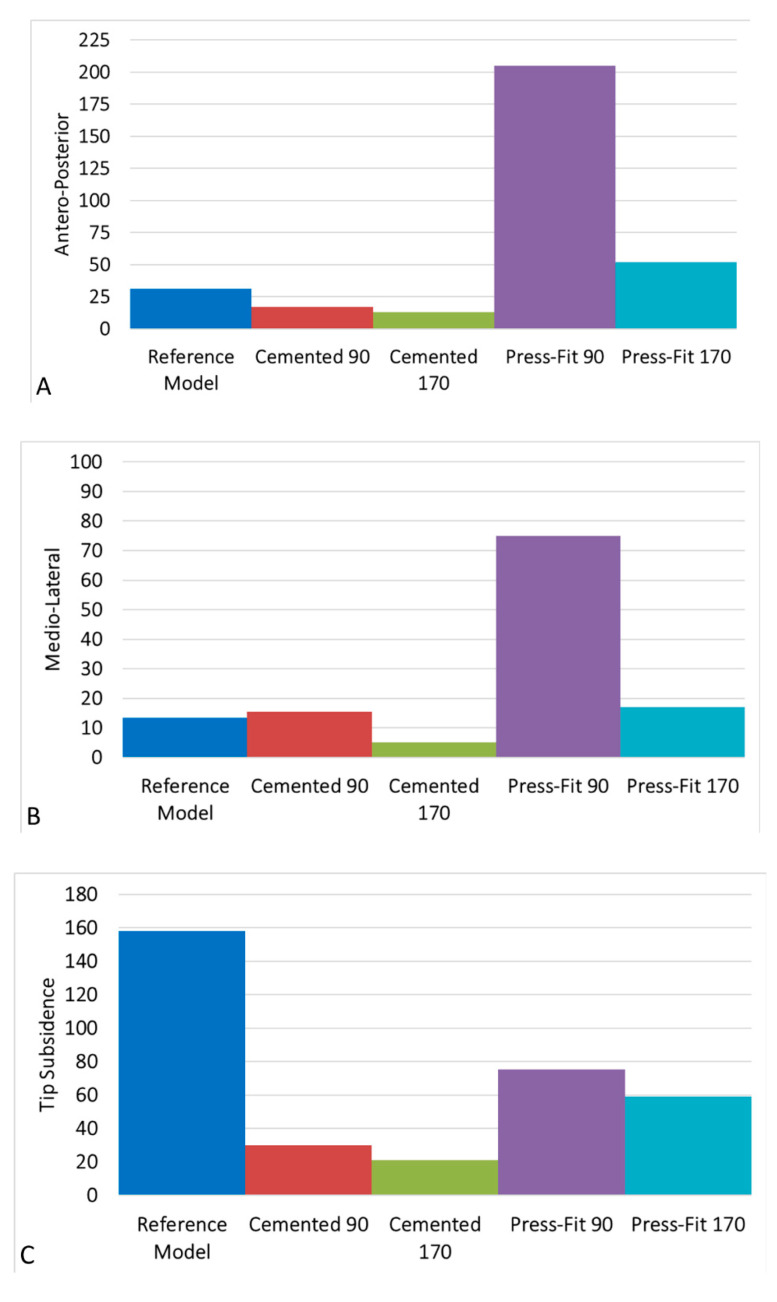
Implant/bone micromotions calculated for all the configurations and compared with reference model ones: antero-posterior (**A**) and medio-lateral (**B**) implant micromotions at the bone-implant interface; stem tip subsidence (**C**).

Concerning implant fixation, Figure 8 reports the antero-posterior and medio-lateral implant micromotions at the bone-implant interface and the subsidence of the stem tip. Coherent with the load conditions, the implant micromotions are higher in the anterior-posterior direction than in the medial-lateral one; in particular, short press-fit stem configurations show the highest micromotions and tip subsidence. The cemented configurations show the lowest micromotions and subsidence and, in detail, a short-cemented stem has lower micromotions compared to a long press-fit stem.

## 4. Discussion

### 4.1. Limitation of the Study

Several assumptions were made in the FE models: firstly, the geometries of the different structures of the developed FE models are based on one anatomy and one implant design; thus, no variation on bone anatomy, bone deformity, or implant mal-alignment was taken in consideration (this approach is nonetheless largely used in modeling clinical situations in the biomechanical research [24,26,28]). Another assumption regards the ligaments, modeled as beams (commonly adopted in literature and in previously validated ligament model [24,26]), and the material models used in this study incorporated several simplifications (as mechanical properties of bony structures and soft tissues were assumed linear elastic and homogeneous), yet still being able to approximate in a proper way the material’s natural behavior.

During an actual surgery, it may be difficult to place a fully press-fit stem into the tibia due to an eccentric canal relative to the tibial plateau; some surgeons may compromise the position of the tibial tray to obtain a truly press-fit stem, if system modularity is not forgiving enough. Furthermore, optimal cementing of the stem is not always achieved in real surgery. However, this study assumed optimal press-fit placement and optimal (4th generation) cementing technique [47] to obtain the data, which was then analyzed.

This study is focused on the biomechanical response of the bone to the implants, depending on design variations of the latter; aspects related to biology and other fields are then excluded from the activity.

### 4.2. Analysis of Femoral Stem

The results of this study demonstrated that the presence of a femoral stem always induces an increase in bone stress. Proportional to stem length increasing, the magnitude of the stress peak and the RF rose, and this latter increases up to 91% for the 280 mm stems; the position of this peak stress is usually situated at the tip of the stem, representing a possible location of failure. These observations are in agreement with the work of Hirschmann et al. [15] (stating that long uncemented stems seemed to generate more pain at the stem tip and that periprosthetic failure might occur near the tip of the stem) and with the studies of Barrack [48,49] (that indicates the mismatch in the elastic modulus between the stem tip and the cortical bone to be a reason for the pain in this area).

Moreover, the present results showed that longer stems might have another potential disadvantage, as they decrease the stress in the distal part of the femur, then becoming responsible for stress shielding and consequently leading to bone structural deterioration [13]. The stress shielding induced by long stems in knee replacements was also observed by Scott et al. [50].

Long stems seemed thus unattractive for TKA and, relying on the present study results, short stems appeared to be more appropriate to reduce the stress shielding effect and the risk of fracture. In addition, in the case of a potential second revision surgery, short stems could be removed, causing less tissue damage if compared to long stems.

Long stems are however often needed to compensate bone loss and instability [15]; hence, the surgeon must weigh up the pros and cons when choosing the right stem for the patient.

Relevant achievement of the present study then consists in the comparison between straight and bowed stems: the femoral anterior bowing has often caused concerns, as it is an influential factor for implant position and it could represent a factor of risk for femoral implant flexion in conventional and revision TKAs [51]. To address this, manufacturers provided bowed stems, affirming that they were designed to fit into the bowed femoral diaphysis and to provide stability. However, to date, no biomechanical analysis has been conducted to assess the influence of bowed stems in terms of stress and strain.

The results of the present study showed that, in 280 mm long stems, bowed ones lead to a reduction of the stress peak at the tip as well as in the overall stress. In the opposite way, in the straight stem, a stress concentration can be approximatively observed at the middle of its length; this result might suggest that this model is unable to fit properly the curvature of the bone, thus engaging excessively the cortex in this area and producing such stress. The 220 mm straight design did not show this effect, mainly because such length was not enough to reach the intra-medullary depth actually requiring a bowing of the stem.

The present study showed that bowing the stem did not only reduce the bone stress but also influenced the RF. This effect was more evident in the 280 mm stem (where the RF was reduced by 43%) than in the 220 mm stem (where the reduction was of 20%). It is suggested that the reduction was lower because the 220 mm bowed stem did not follow the curvature of the bone as well as the 280 mm stem did.

Relying upon these results, it may thus seem obvious to recommend using bowed stems if a long stem is needed, but caution needs to be taken: the proper alignment of a bowed stem is indeed more difficult to reach, as bone curvature is different in all patients.

The morphological analysis then showed that the presence of a slot is able to reduce the RF: the decrease varied from 8% (160 mm solid stem) to 37% (280 mm bowed solid stem), results easy to foresee since the stiffness of the press-fit stem is substantially reduced by the presence of the slot. This feature also appears to reduce the pain at the stem tip; although a multifactorial phenomenon, thigh pain is indeed believed to be due to locally higher pressures and stresses [29,52], and these results are in agreement with the ones found by Hirschmann et al. [15] and Barrack et al. [48,49], suggesting that the slotted stem causes less “stem-end pain” than the solid one (in detail, Barrack et al. found that 9.8% of patients with slotted stems experienced stem tip pain, whereas with un-slotted stems, the percentage rose up to 32%).

Often used to enhance fixation [15], flutes are very common features but until now, no study has yet investigated the impact of those latter on bone stress and RF. The simulations performed in the present study showed that the flutes act as stress risers, increasing the risk of fracture in each model incorporating them (i.e., RF increased up to 66% for the 280 mm bowed stem). However, these values depend on the profile: for each design, indeed, the engagement between flutes and bone was different and thus the impact of the flutes may vary depending on their penetration in the cortical bone.

The RF in fluted configurations then happens to reach quite high values for long stems; whereas the limit considered as acceptable is set to 1, in the 280 mm straight fluted stem, it reached the critical value of 1.02 (although it is to be kept in consideration that this configuration was not a conventional one and was designed with the sole purpose to serve as a comparison for the 280 mm bowed stem). It is then to be considered that only one type of flute was designed and analyzed in the present study, whereas several different typologies currently exist, each of them with a different influence.

Based upon the present study, it is then suggested not to apply flutes in patients with a high risk of bone fracture and, if absolutely needed, it is recommended to combine them with a slot in order to reduce the stiffness of the stem.

The stress shielding shown in the 220 mm stem was also observed in the study performed by Oldani et al. [53], which aimed to assess the impact of medical device material properties on the bone; this effect could also be worsened by the potential lack of osteointegration in Cobalt-chrome (as demonstrated by Plecko et al., comparing it to the other metals [54]).

Gililland et al. [18] published clinical and radiographic outcomes of cemented and diaphyseal, engaging cementless stems in aseptic revision TKA, and reporting a difference based on bones quality; a significantly lower radiographic failure rate was observed in good quality bone (3%) when compared to poor quality bone (33%). Similarly, the present study showed that osteoporosis seems to weaken the bone rather than exposing it to fractures (the RF indeed increased in all studied stems). This increase was already observed with the reference model (RF rise of 44%), but it reached even higher values with the long stems (97% for the 280 mm bowed and fluted stems).

Hence, great care should be taken when dealing with patients suffering from osteoporosis and the choice of the implant must be done considering this important factor.

### 4.3. Analysis of Tibial Stem

Compressive stress in the proximal tibia results in a reduction of 7.3% on the medial side when using a long-cemented stem instead of a short one; similarly, using a long press-fit stem instead of a short one makes the stress decrease by 62%. This trend is in agreement with the study carried out by Jazrawi et al. [55], reporting that the proximal tibial strain decreased as the stem length increased. The comparison between stemmed models and the reference model highlighted a general increase in the stress in the proximal tibia, starting from 1.05% for the cemented short stem up to 188% for the press-fit short stem. In the long-cemented stem and the press-fit one, instead, 17.88% and 36.65% were found, respectively. The enormous increment for the press-fit short stem can be traced back to the presence of a slot: the bone undergoes the load closer to the tibial tray because it is not long enough to distribute the load further, as its stiffness decreases where the slot begins. Being aware of this, special care should be used if willing to implant a press-fit short-slotted stem. Reducing proximal stress is useful in the presence of relevant proximal bony defects but may lead to proximal bone resorption and thus contribute to tibial component loosening; proximal stress is then necessary to prevent bone loss, but it should, however, be kept in a reasonable range in order to avoid fractures.

Results are also in agreement with the study by Reilly et al., reporting that a long- cemented stem allows direct load transfer to the cortex at the tip of the stem [56]; focusing on maximal average compressive stress, part of the outcome is then in agreement with El-Zayat et al. [47] (since long stems result in having higher maximal compressive stress compared to short ones), but the values obtained in the present study argue with the above-mentioned ones as the maximal compressive stress for press-fit short stems returned lower than for long-cemented stems (again, this can be attributed to the reduction in the stiffness of the stem). Continuing the comparison with El-Zayat study, press-fit unslotted stems appear to have higher average maximal stress than press-fit slotted stems, even with the latter undergoing heavier loading conditions.

According to the available literature, the long-cemented stem presents the maximal average compressive stress at the stem tip, whereas the other stemmed models do not; in particular, press-fit models present this maximum in the region where the teeth–bone engagement begins. These findings were predictable since the stiffness of the press-fit stem is substantially reduced by the presence of the slot. Diminishing load transfer at the stem tip results in a minor incidence of end of stem pain in subjects with TKA.

Stems influence on micromovements has been studied by Completo et al. [17], Jazrawi et al. [55], and Stern et al. [57]; the latter used 30 cadaveric tibias and showed that longer stems were associated with higher micromotions and that the same behavior was present in press-fit stems. Using three synthetic tibias, Completo reported that the displacements were reduced for the press-fit stem by 19% and for cemented one by 23% compared to a standard model without stem. Employing six frozen tibias from human cadavers, Jazrawi instead concluded that tibial tray micromotions decreased with the increase of press-fit stem length and diameter; he also found that cemented stems showed lower motions than uncemented stems.

The above-mentioned papers agree with the results of the present study, proving that the lowest micromovements at the bone-tibial tray interface and at the stem tip are found in long-cemented models (up to more than three times). Tibial tray-bone displacements reduce by 16.8% and 62.2%, respectively, for short- and long-cemented stems in comparison to the reference model. Opposingly, an increase in press-fit stems can be noted if compared to the reference model, and this can be attributed to the presence of the slot.

It should be remarked that in this FEA, bony ingrowth was not considered, as only the early phase after the TKA was simulated; sometime after the surgical procedure, the bone can indeed grow inside the slot and thus it can lead to a better fixing of the implant itself.

Stem tip displacement appears to be related to the fixation technique, as it is higher for the press-fit than for the cemented stems; compared to the reference model, axial stem tip motion diminishes by 69% for the short press-fit stem, by 76.4% for the long press-fit stem, by 76.8% for the short-cemented stem, and by 79.19% for the long-cemented stem. A short-cemented stem and long press-fit stem have approximately the same behavior, so they allow for reaching an equivalent stability.

From the graphical overview of the contact pressure, the short press-fit model presented more pressure around the tibial tray pegs; the material applied for this region is the 3 mm thick cement layer and the underlying cancellous bone, so this is the reason why the short press-fit model reduces its axial displacement less than other models.

Results obtained for the short press-fit slotted model suggest that manufacturing this stem only in the unslotted configuration could be an optimal choice, in order to maintain lower stresses in the proximal tibia.

Furthermore, as it has already been said above, a short-cemented stem presents the same displacement as a long press-fit stem, so it should be recommended to implant a short-cemented stem rather than weaken the bone even more with a long one.

## 5. Conclusions

The goal of the present study was to provide a biomechanical overview of the different effects corresponding to the several stem features that can be applied to provide stability in the femur and in the tibia during a revision TKA. The results demonstrated that anatomical shapes and slots are able to reduce bone stress and the risk of fracture, whereas flutes did the opposite. No relevant differences were found in this purview between cemented and press-fit stems. Cemented tibial stems have proven to reduce antero-posterior micromotions, playing an important role in preventing implant loosening.

## Data Availability

Not applicable.

## References

[1] Indelli P.F., Giori N., Maloney W. (2015). Level of constraint in revision knee arthroplasty. Curr. Rev. Musculoskelet. Med..

[2] Sharkey P.F., Hozack W.J., Rothman R.H., Shastri S., Jacoby S.M. (2002). Insall award paper: Why are total knee arthroplasty failing today?. Clin. Orthop. Relat. Res..

[3] Sharkey P.F., Lichstein P.M., Shen C., Tokarski A.T., Parvizi J. (2014). Why Are Total Knee Arthroplasties Failing Today—Has Anything Changed After 10 Years?. J. Arthroplast..

[4] Kurtz S., Ong K., Lau E., Mowat F., Halpern M. (2007). Projections of primary and revision hip and knee arthroplasty in the United States from 2005 to 2030. J. Bone Jt. Surg. Am..

[5] Hossain F., Patel S., Haddad F.S. (2010). Midterm assessment of causes and results of revision total knee arthroplasty. Clin. Orthop. Relat. Res..

[6] Mabry T.M., Vessely M.B., Schleck C.D., Harmsen W.S., Berry D.J. (2007). Revision total knee arthroplasty with modular cemented stems: Long-term follow-up. J. Arthroplast..

[7] Peters C.L., Erickson J., Kloepper R.G., Mohr R.A. (2005). Revision total knee arthroplasty with modular components inserted with metaphyseal cement and stems without cement. J. Arthroplast..

[8] Fehring T.K., Odum S., Olekson C., Griffin W.L., Mason J.B., McCoy T.H. (2003). Stem fixation in revision total knee arthroplasty: A comparative analysis. Clin. Orthop. Relat. Res..

[9] Shannon B.D., Klassen J.F., Rand J.A., Berry D.J., Trousdale R.T. (2003). Revision total knee arthroplasty with cemented components and uncemented intramedullary stems. J. Arthroplast..

[10] Sierra R.J., Cooney W.P., Pagnano M.W., Trousdale R.T., Rand J.A. (2004). Reoperations after 3200 revision TKAs: Rates, etiology, and lessons learned. Clin. Orthop. Relat. Res..

[11] Whaley A.L., Trousdale R.T., Rand J.A., Hanssen A.D. (2003). Cemented long-stem revision total knee arthroplasty. J. Arthroplast..

[12] Wood G.C., Naudie D.D., MacDonald S.J., McCalden R.W., Bourne R.B. (2009). Results of press-fit stems in revision knee arthroplasties. Clin. Orthop. Relat. Res..

[13] Grover K., Lin L., Hu M., Muir J., Qin Y.X. (2016). Spatial distribution and remodeling of elastic modulus of bone in micro-regime as prediction of early stage osteoporosis. J. Biomech..

[14] Fehring T.K., Bono J.V., Scott R.D. (2005). Use of Stems in Revision Total Knee Arthroplasty. Revision Total Knee Arthroplasty.

[15] Hirschmann M.T., Becker R. (2015). The Unhappy Total Knee Replacement.

[16] Brand M.G., Daley R.J., Ewald F.C., Scott R.D. (1989). Tibial tray augmentation with modular metal wedges for tibial bone stock deficiency. Clin. Orthop. Relat. Res..

[17] Completo A., Simões J.A., Fonseca F., Oliveira M. (2008). The Influence of Different Tibial Stem Designs in Load Sharing and Stability at the Cement-Bone Interface in Revision TKA. Knee.

[18] Gililland J.M., Gaffney C.J., Odum S.M., Fehring T.K., Peters C.L., Beaver W.B. (2014). Clinical & Radiographic Outcomes of Cemented vs. Diaphyseal Engaging Cementless Stems in Aseptic Revision TKA. J. Arthroplast..

[19] Mountney J., Wilson D.R., Paice M., Masri B.A., Greidanus N.V. (2008). The Effect of an Augmentation Patella Prosthesis Versus Patelloplasty on Revision Patellar Kinematics and Quadriceps Tendon Force: An Ex Vivo Study. J. Arthroplast..

[20] Pagnano M.W., Trousdale R.T., Rand J.A. (1995). Tibial Wedge Augmentation for Bone Deficiency in Total Knee Arthroplasty: A Follow-up Study. Clin. Orthop. Relat. Res..

[21] Sarmah S.S., Patel S., Reading G., El-Husseiny M., Douglas S., Haddad F.S. (2012). Periprosthetic fractures around total knee arthroplasty. Ann. R. Coll. Surg. Engl..

[22] Bori E., Armaroli F., Innocenti B. (2021). Biomechanical analysis of femoral stems in hinged total knee arthroplasty in physiological and osteoporotic bone. Comput. Methods Programs Biomed..

[23] Innocenti B., Bori E., Armaroli F., Schlager B., Jonas R., Wilke H.-J., Galbusera F., Innocenti B., Galbusera F. (2022). The use of computational models in orthopedic biomechanical research. Human Orthopaedic Biomechanics.

[24] Pianigiani S., Innocenti B., Stewart J. (2015). The use of finite element modeling to improve biomechanical research on knee prosthesis. New Developments in Knee Prosthesis Research.

[25] Andreani L., Pianigiani S., Bori E., Lisanti M., Innocenti B. (2019). Analysis of biomechanical differences between condylar constrained knee and rotating hinged implants: A numerical study. J. Arthroplast..

[26] Labey L., van Lenthe G.H., Sloten J.V., Catani F. (2014). Load sharing and ligament strains in balanced, overstuffed and understuffed UKA. A validated finite element analysis. J. Arthroplast..

[27] McNamara B.P., Cristofolini L., Toni A., Taylor D. (1997). Relationship between bone-prosthesis bonding and load transfer in total hip reconstruction. J. Biomech..

[28] Innocenti B., Fekete G., Pianigiani S. (2018). Biomechanical Analysis of Augments in Revision Total Knee Arthroplasty. J. Biomech. Eng..

[29] Rastetter B.R., Wright S.J., Gheduzzi S., Miles A.W., Clift S.E. (2016). The influence of tibial component malalignment on bone strain in revision total knee replacement. Proc. H Inst. Mech. Eng..

[30] Soenen M., Baracchi M., De Corte R., Labey L., Innocenti B. (2013). Stemmed TKA in a femur with a total hip arthroplasty. Is there a safe distance between the stem tips?. J. Arthroplast..

[31] Viceconti M., Casali M., Massari B., Cristofolini L., Bassini S., Toni A. (1996). The ‘standardized femur program’. Proposal for a reference geometry to be used for the creation of finite element models of the femur. J. Biomech..

[32] Innocenti B., Pianigiani S., Labey L., Victor J., Bellemans J. (2011). Contact Forces in Several TKA Designs During Squatting: A Numerical Sensitivity Analysis. J. Biomech..

[33] Pianigiani S., Chevalier Y., Labey L., Pascale V., Innocenti B. (2012). Tibio-Femoral Kinematics in Different Total Knee Arthroplasty Designs During a Loaded Squat: A Numerical Sensitivity Study. J. Biomech..

[34] Victor J., Van Doninck D., Labey L., Innocenti B., Parizel P.M., Bellemans J. (2009). How Precise Can Bony Landmarks Be Determined on a CT Scan of the Knee?. Knee.

[35] Innocenti B., Bellemans J., Catani F. (2016). Deviations from Optimal Alignment in TKA: Is There a Biomechanical Difference Between Femoral or Tibial Component Alignment?. J. Arthroplast..

[36] Parratte S., Pagnano M.W., Trousdale R.T., Berry D.J. (2010). Effect of Postoperative Mechanical Axis Alignment on the Fifteen-Year Survival of Modern, Cemented Total Knee Replacements. J. Bone Jt. Surg. Am..

[37] Ingrassia T., Nalbone L., Nigrelli V., Tumino D., Ricotta V. (2013). Finite element analysis of two total knee prostheses. Int. J. Interact. Des. Manuf..

[38] Rho Y.J., Kuhn-Spearing L., Zioupos P. (1998). Mechanical properties and the hierarchical structure of bone. Med. Eng. Phys..

[39] Sarathi Kopparti P., Lewis G. (2007). Influence of three variables on the stresses in a three-dimensional model of a proximal tibia-total knee implant construct. Biomed. Mater. Eng..

[40] Brihault J., Navacchia A., Pianigiani S., Labey L., De Corte R., Pascale V., Innocenti B. (2016). All-polyethylene tibial components generate higher stress and micromotions than metal-backed tibial components in total knee arthroplasty. Knee Surg. Sports Traumatol. Arthrosc..

[41] Kayabasi O., Ekici B. (2007). The effects of static, dynamic and fatigue behavior on three-dimensional shape optimization of hip prosthesis by finite element method. Mater. Des..

[42] Innocenti B., Pianigiani S., Ramundo G., Thienpont E. (2017). Biomechanical Effects of Different Varus and Valgus Alignments in Medial Unicompartmental Knee Arthroplasty. J. Arthroplast..

[43] Galbusera F., Freutel M., Dürselen L., D’Aiuto M., Croce D., Villa T., Sansone V., Innocenti B. (2014). Material Models and Properties in the Finite Element Analysis of Knee Ligaments: A Literature Review. Front. Bioeng. Biotechnol..

[44] Vaninbroukx M., Labey L., Innocenti B., Bellemans J. (2009). Cementing the femoral component in total arthroplasty: Which technique is the best?. Knee.

[45] Pianigiani S., Labey L., Pascale W., Innocenti B. (2016). Knee Kinetics and Kinematics: What Are the Effects of TKA Malconfigurations?. Knee Surg. Sports Traumatol. Arthrosc..

[46] Vanlommel J., Luyckx J.P., Labey L., Innocenti B., De Corte R., Bellemans J. (2011). Cementing the Tibial Component in Total Knee Arthroplasty: Which Technique Is the Best?. J. Arthroplast..

[47] El-Zayat B.F., Heyse T.J., Fanciullacci N., Labey L., Fuchs-Winkelmann S., Innocenti B. (2015). Fixation techniques and stem dimensions in hinged total knee arthroplasty: A finite element study. Arch. Orthop. Trauma Surg..

[48] Barrack R.L., Rorabeck C., Burt M., Sawhney J. (1999). Pain at the end of the stem after revision total knee arthroplasty. Clin. Orthop. Relat. Res..

[49] Barrack R.L., Stanley T., Burt M., Hopkins S. (2004). The effect of stem design on end-of-stem pain in revision total knee arthroplasty. J. Arthroplast..

[50] Scott C.E., Biant L.C. (2012). The role of the design of tibial components and stems in knee replacement. J. Bone Joint Surg. Br..

[51] Ko J.H., Han C.D., Shin K.H., Nguku L., Yang I.H., Lee W.S., Kim K.I., Park K.K. (2016). Femur bowing could be a risk factor for implant flexion in conventional total knee arthroplasty and notching in navigated total knee arthroplasty. Knee Surg. Sports Traumatol. Arthrosc..

[52] Kim Y.H., Kwon O.S., Kim K. (2008). Analysis of biomechanical effect of stem-end design in revision TKA using Digital Korean model. Clin. Biomech..

[53] Oldani C., Dominguez A., Fokter S.K. (2012). Titanium as a Biomaterial for Implants. Recent Advances in Arthroplasty.

[54] Plecko M., Sievert C., Andermatt D., Frigg R., Kronen P., Klein K., Stübinger S., Nuss K., Bürki A., Ferguson S. (2012). Osseointegration and biocompatibility of different metal implants—A comparative experimental investigation in sheep. BMC Musculoskelet. Disord..

[55] Jazrawi L.M., Bai B., Kummer F.J., Hiebert R., Stuchin S.A. (2001). The effect of stem modularity and mode of fixation on tibial component stability in revision total knee arthroplasty. J. Arthroplast..

[56] Reilly D., Walker P.S., Ben-Dov M., Ewald F.C. (1982). Effects of tibial components on load transfer in the upper tibia. Clin. Orthop. Relat. Res..

[57] Stern S.H., Wills D., Gilbert J.L. (1997). The effect of tibial stem design on component micromotion in knee arthroplasty. Clin. Orthop. Relat. Res..

